# Performance of Combined Woven Roving and Mat Glass-Fiber Reinforced Polymer Composites Under Absorption Tower Lifting Loads

**DOI:** 10.3390/polym16202937

**Published:** 2024-10-19

**Authors:** Víctor Tuninetti, Matías Mariqueo

**Affiliations:** Department of Mechanical Engineering, Universidad de La Frontera, Temuco 4811230, Chile; m.mariqueo01@ufromail.cl

**Keywords:** glass-fiber reinforced polymer, finite element analysis, absorption tower, lifting operations

## Abstract

This study investigates the structural integrity of a glass-fiber reinforced polymer absorption tower during lifting operations, evaluating factors of safety and stress distribution for both horizontal and vertical scenarios. A key focus is the comparative analysis of surface and volumetric meshing techniques in finite element modeling. Results demonstrate that surface models achieve comparable stress predictions to computationally intensive volumetric models, significantly reducing computational demands without compromising accuracy. For instance, stress at the flange edge with holes was accurately captured using a surface model with 5675 elements (12.79 MPa), yielding similar results to a volumetric model requiring over 94,000 elements (13.37 MPa). Similar computational efficiency and agreement between modeling approaches were observed at the packing support ring-shell joint. Finite element analysis employing Hashin’s failure criterion, informed by industry-standard experimental data, revealed safety factors ranging from 1.9 to 2.5 for horizontal lifting and four for vertical lifting. These safety factors indicate sufficient margins for safe operation. While these findings support the feasibility of both lifting methods, further investigation is recommended to address the lower safety factors observed in specific horizontal lifting scenarios. A comprehensive assessment incorporating industry standards, dynamic load effects, and potential mitigation strategies is crucial to ensure the long-term structural integrity of the GFRP absorption tower.

## 1. Introduction

Fiber-reinforced polymer composites are commonly used in industry due to their properties, such as their strength-to-weight ratio, corrosion resistance, and ease of fabrication [1,2]. However, it is important to investigate the structural performance of these composite components when subjected to lifting operations for maintenance purposes. Hence, assessing the structural feasibility of lifting operations is critical when designing tower sections to prevent damage to structural elements and maintain operational safety.

This study evaluates the feasibility of lifting or hoisting sections of a glass-fiber reinforced polymer gas absorption tower while ensuring the structural integrity and safety of the lift. The tower is located within an industrial production facility, and the lifting operation must avoid any impacts that could cause structural damage [3]. To warn and reduce potential hazards, a real-time safety warning system using a deep learning technique could also be used [4]. In order to study the disassembly and lifting maneuver, the full mechanical properties of FRP [5,6] including manufacturing conditions [7] must be taken into account.

The strength of glass-fiber reinforced polymer (GFRP) composites can be characterized through various methods. Material databases provide information on the mechanical properties of GFRP, such as tensile, compressive, and flexural strength, which can be used in the design and analysis of GFRP structures [6,8,9]. Experimental testing, including tensile, compression, and flexural tests, can directly measure the mechanical properties of the specific GFRP material used in the absorption tower [10]. Additionally, based on different factors like fiber volume fraction, fiber orientation, and manufacturing process, artificial neural network models can be developed to predict the mechanical behavior of GFRP [11]. To further understand the long-term performance of GFRP, additional testing should be conducted to evaluate the degradation of material properties due to exposure to environmental factors such as ultraviolet radiation, moisture, temperature extremes, and chemical agents, which can impact the durability and structural integrity of the GFRP components [12].

In this research, the tower sections are numerically studied using adequate damage models and material properties from industry standards. The towers are structurally joined by flanges and fastened by bolts [13,14,15]. These same joint areas will be used when joining the hoisting elements, and consequently during the maneuver or lifting process, the FRP section could suffer tensile, combined [16,17], and shear stresses [18,19,20], depending on the instant or stage of the process. In addition, the tower shows manways and flanges in its shell required for functionality [21,22], which during hoisting may present stress concentrators that must be carefully considered [23] for the structural analysis. In addition, the absorption tower is equipped with ceramic packing, presenting an additional load to the structure [24,25]. Given the anisotropic nature of reinforced composite materials, the analysis should consider an orthotropic failure criterion [2]. Neglecting this could lead to an underestimation of the material response and overestimation of the safety factor, increasing the risk of failure [26].

The structural integrity of the system during lifting is performed using the computational finite element method (FEM) from ANSYS Mechanical Composite Prepost (ACP). The largest number of elements that compose the system and the additional loads associated with the ceramic packing are also considered for the critical scenarios. The numerical analysis of the lifting operations for the GFRP tower sections, using Hashin’s failure criterion and industry-standard-informed experimental data, leads to factors of safety ranging from 1.9 to 2.5 for horizontal lifting and a factor of safety of 4.0 for vertical lifting.

The digital models, design steps, and analysis of results provide important recommendations for the design of lifting operations for glass-fiber reinforced polymer (GFRP) composite absorption towers.

## 2. Structural Evaluation Process of the Hoisting Operation Using a Computational Tool

**Figure 1:**
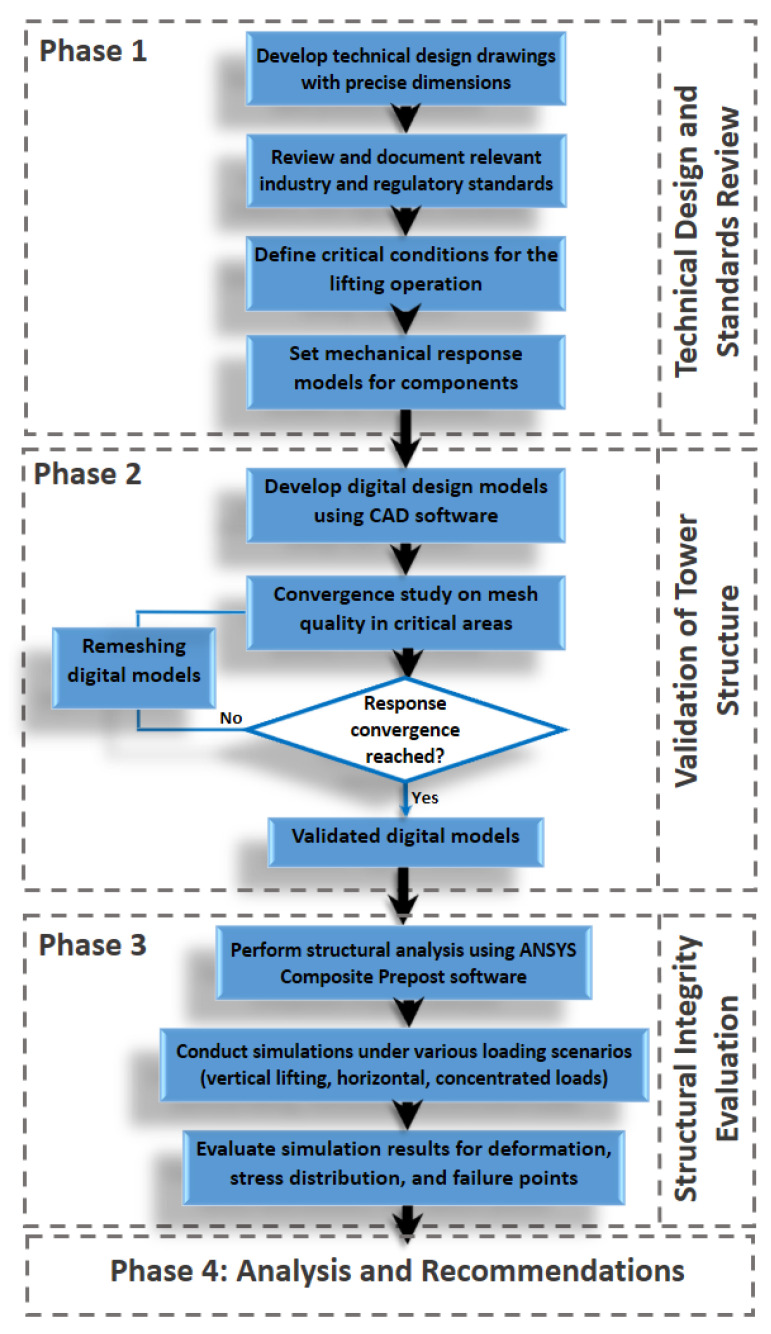
Flowchart of the four-step process design for the structural assessment of the hoisting operation of the GFRP absorption tower using a computational tool.

This work was carried out in four phases (Figure 1). First, the technical design drawings with dimensions were established, and the relevant standards were reviewed. Additionally, the critical conditions for the lifting operation were defined, and mechanical response models of the components and their characteristic parameters were created. In the second phase, the structure of the absorption tower sections was validated by developing digital design models and studying the convergence of meshes in critical sections and areas of interest. The structural integrity of the tower sections was then evaluated using Mechanical Composite PrePost (ACP) ANSYS software (student 2023 R2 version). Finally, in the concluding analysis and recommendations stage, elevation guidelines were established based on numerical calculations to ensure the safe operation of the system in terms of structural integrity.

### 2.1. System Components

The GFRP tower has an approximate height of 15 m; it is composed of several sections of approximately 3 m. This absorption tower is used in a purification process that contains ceramic packing in its interior, required to improve the absorption efficiency; the weight of these elements must be considered because they are significant to the tower. The tower material, GFRP, being a composite material, could have adverse behaviors when subjected to particular loads such as lifting maneuvers. The main components are detailed in the following sections.

#### 2.1.1. Dimensions of the Absorption Tower Sections

**Figure 2:**
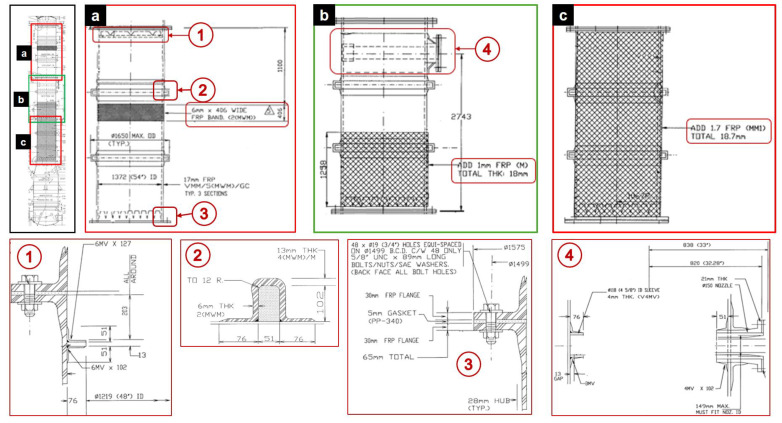
Absorption tower showing details of the analyzed intermediate sections (**a**–**c**).

The gas absorption tower consists of five sections of GFRP: the base, three intermediate sections, and the top (Figure 2). To perform maintenance on the tower it is necessary to disassemble and lift each section separately. Only the three sections of the intermediate zone of the GFRP tower will be considered for this study as they are considered critical for the operation (Figure 2a–c). The typical dimensions of the intermediate sections of the gas absorption tower are detailed below.

- Each section has a length of 3048 mm, an inside diameter of 1372 mm, a minimum wall thickness of 17 mm, and an outside flange diameter of 1575 mm.
- The upper and lower flanges of each section have 48 equidistant 19 mm diameter holes with a bolt circle diameter of 1499 mm and a wall thickness of 30 mm.
- Each of the GFRP tower sections has 38 mm rings located in the lower areas. The lower ring supports 5000 kg of ceramic packing.
- All sections have two structural reinforcements of variable thickness from 6 to 13 mm.

The distinctive features of each intermediate section must be considered for the model. Intermediate section (1) (Figure 2a) is located in the upper zone, section (2) (Figure 2b) in the middle zone, and section (3) (Figure 2c) in the lower zone. The distinctive features of each section are presented below.

- Section (1): It has a reinforcement band 406 mm long and 6 mm thick, located 1100 mm from the upper edge. In addition, it has a 10 mm thick inner ring that supports a demister of the absorption tower.
- Section (2): It has two nozzles, an inner nozzle with a diameter of 118 mm and a thickness of 4 mm and a through nozzle with a diameter of 150 mm and a thickness of 21 mm, which support the liquid distributor. These nozzles are attached to the section by bandages, with the inner bandages being 4 mm thick and the outer bandage 27 mm thick. Additionally, this section has a 1 mm thick, 1258 mm long overlay on the bottom flange.
- Section (3): This section has a 1.7 mm thick coating on its shell.

#### 2.1.2. Fibers and Laminate

The gas absorption tower is designed from composite materials, specifically GFRP. The typical manufacturing process for this type of tower is by the filament winding method. Structurally, a layered system is formed. The gas absorption tower consists of three types of layers: two structural and one chemical barrier layer. For the study of the hoisting or mechanical lifting of the tower, only the structural layers will be considered. The structural layers consist of two types of resin-bonded fibers: Mat fiber (M) and Woven Roving fiber (W). Each tower component is made up of a sequence of “MWM” laminates. The fiber angulation considered in this study corresponds to ±55° with respect to the *Y*-axis. Fiber angulation at ±55° has been the subject of study and demonstrates favorable mechanical performance in tubular specimens during tensile testing [27]. In addition, progressive failure studies have also shown positive responses for this fiber angulation [28].

The lamination sequence of the tower sections is detailed as follows: Mat at 55°, Woven Roving at −55°, Mat at 55°, Mat at −55°, Woven Roving at 55°, and finally, Mat at −55°. The thickness considered for Woven Roving fiber is 1 mm and for Mat fiber is 0.8 mm. Table 1 presents the lamination by zone of the absorption tower sections.

#### 2.1.3. Woven Roving and Mat Glass-Fiber Reinforced Polymer Composite

Woven Roving is a fabric which is manufactured by interlacing fiber filaments in two perpendicular directions. This fabric is characterized by good strength and durability. Mat fibers are a nonwoven fibrous reinforcement material composed of randomly distributed fiber filaments. It is widely used in the manufacture of composite structures. Table 2 presents the mechanical properties of Woven Roving and Mat glass-fiber reinforced polymeric composite material based on the ASME RTP-1 standard and ANSYS library.

### 2.2. Critical Conditions and Parameters for the Lifting Maneuver

The lifting maneuver involves mobilizing each section of the absorption tower individually. The study seeks to validate the lifting of these sections for both vertical and tandem lifting. The critical conditions for the lifting maneuver are detailed below.

#### 2.2.1. Loading Conditions

The loads include the self-weight of the tower sections and the weight of the ceramic packing. The weight of the tower sections is estimated to be 600 [kg]; this weight was considered according to the density of the Mat/Woven Roving/Mat (MWM) sequence of the GFRP laminates described in Section 2.1.2 and Modeled in Section 2.4. The weight of the ceramic packing is 5000 kg; this value was extracted from the technical drawings. These loads will be distributed according to the type of lifting (vertical or tandem). Dynamic loads, such as vibrations or impacts, will be considered negligible for the simulations, since no impacts are foreseen during the maneuver.

#### 2.2.2. Constraints and Supports

The lifting elements will be bolted to the tower sections using the existing holes in the flanges. These holes will be displacement constrained in the corresponding direction of the lifting condition: vertical or tandem. The ceramic packing, installed on a circular support, will be secured within a GFRP ring integrated into the structure of each tower section. 

#### 2.2.3. Environmental Conditions

Environmental conditions, such as wind loads, shall be considered negligible during the maneuver, since the maneuver must be carried out in the absence of strong wind. Also, abrupt changes in ambient temperature shall be minimized, ensuring that the temperature does not vary suddenly during the lifting.

### 2.3. Generation of Virtual Models of the Absorption Tower

**Figure 3:**
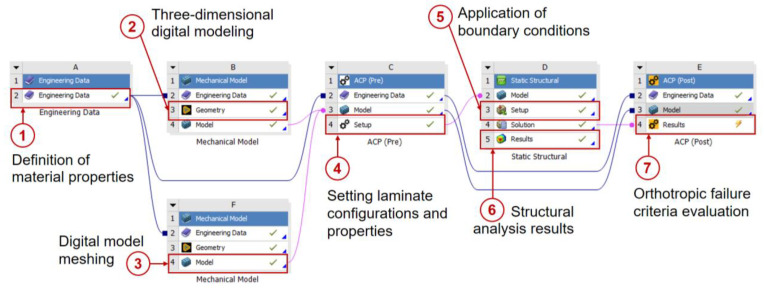
Stages of structural integrity analysis for the GFRP absorption tower sections using ANSYS Workbench software.

Modeling in ANSYS ACP PREPOST software was chosen to carry out a structural integrity evaluation of the gas absorption tower sections during lifting operations using the following steps (Figure 3):

1. Engineering Data: In this module, the mechanical properties of the different materials to be used in the simulation are entered. Parameters such as the modulus of elasticity, Poisson’s ratio, and density, among others, are defined here to ensure that the analysis reflects the real characteristics of the materials.
2. Mechanical Model: In this module, the geometries of the tower sections are imported and defined. Subsequently, an appropriate meshing is applied to the needs of the analysis, which allows discretizing the geometry for its structural evaluation.
3. ACP (Pre): This module is used to define the properties of the laminates in the imported geometries. The laminate layups are managed and the properties of the composite materials are assigned to the different tower sections.
4. Static Structural: In this module, the boundary conditions are applied and the loads corresponding to the tower sections are introduced. A static analysis is performed to evaluate how the materials respond to the applied loading conditions.
5. ACP (Post): This module allows a detailed analysis of the failure criteria for composite materials. Simulation results are examined to determine the behavior of the composite material under the applied loading conditions, identifying possible failure points.

#### Conversion of Technical Drawings to Digital Models Using Autodesk Inventor

For the process of converting the details of the technical drawings to digital models, Autodesk Inventor software (student version) was used. Plate element designs were created using manly revolutions and extrusion functions. The base model was created by assembling two geometries which are a base section and typical reinforcement. The details of these geometries are presented below.

- The base section has a height of 3048 mm, with an inside diameter of 1372 mm and an outside flange diameter of 1575 mm. In addition, it includes a hole circle with a radius of 749.5 mm in each flange. Each hole circle has 48 holes each with a radius of 9.5 mm. The base section also features a 13 mm notch radius at its flanged edges.
- In the lower zone of the base section, there is a cut in the shell at 203 mm from the lower edge, which represents an overthickness. Additionally, in the upper zone, the same cut is present in relation to the upper edge, except in Section 1, where it varies at 106 mm in the upper zone. At 51 mm from the lower edge of the base section is the support ring for the ceramic packing, which has an inner radius of 635 mm and an outer radius of 686 mm.
- The typical reinforcement has the shape of a circular band with a radius of 686 mm and a height of 203 mm. In its middle zone, it has a projection with an outer radius of 788 mm and a height of 51 mm. In addition, the corners of the reinforcement are rounded with a notch radius of 12 mm.

Starting from the base model, three sections of the gas absorption tower were designed, each with specific characteristics described below.

**Figure 4:**
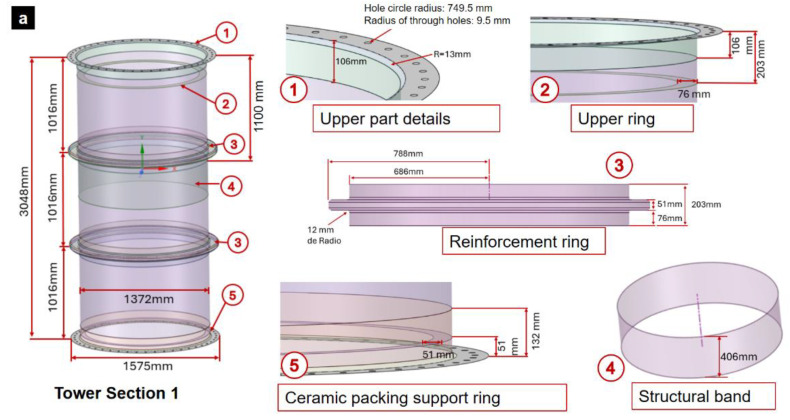

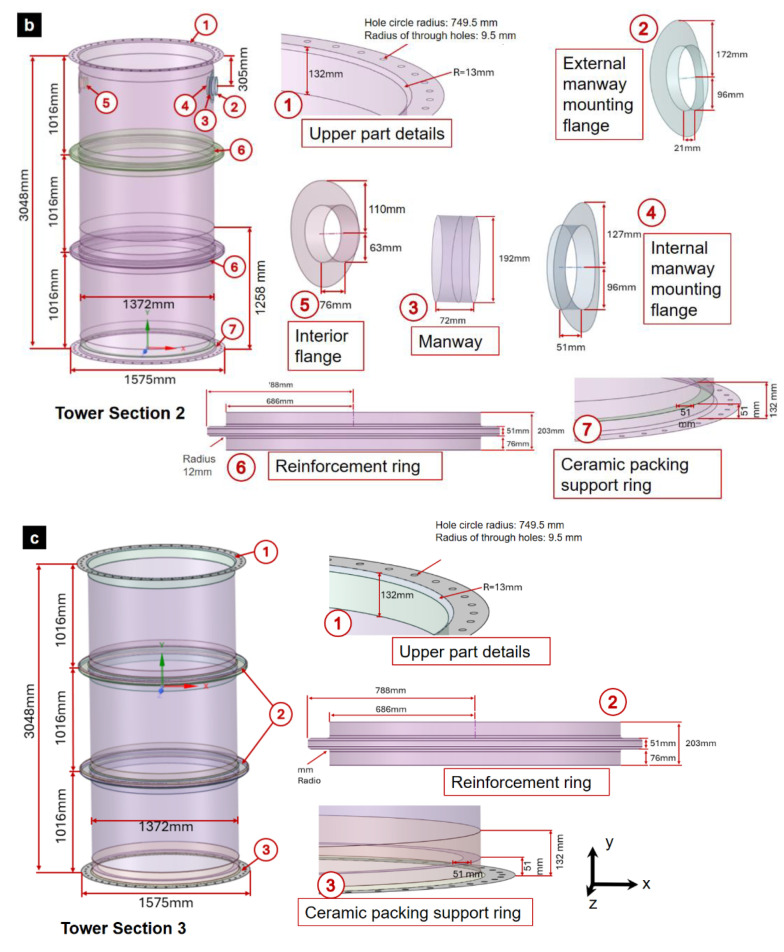
Digital modeling of intermediate GFRP tower: (**a**) section (1), (**b**) section (2), and (**c**) section (3).

- Section (1): This section features a 406 mm wide structural band located 1100 mm from the top edge. In addition, this section has an upper support ring for the demister, located 203 mm from the top edge. This ring has an inner radius of 610 mm and an outer radius of 686 mm (Figure 4a).
- Section (2): This section has a manway with a diameter of 96 mm and a length of 72 mm. The manway is attached to the shell section by an outer and inner mounting flange. The outer flange has an inner radius of 96 mm, an outer radius of 172 mm and an elevation of 21 mm. The inner flange has an inner radius of 96 mm, an outer radius of 127 mm and an elevation of 51 mm. In addition, this section has an interior flange with an inner radius of 63 mm, outer radius of 110 mm, and a length of 76 mm (Figure 4b).
- Section (3): This section does not present differentiating details with respect to the base model; in fact, the base model is based on this section (Figure 4c).

### 2.4. Modeling the Laminate of the Gas Absorption Tower Sections

The structural laminate follows the MWM sequence, which corresponds to Mat, Woven Roving, and Mat fibers, respectively. The drawings of the designs do not specify angulations of the fibers; however, an angulation of ±55° with respect to the *Y*-axis will be used. The lamination sequence is detailed as follows: Mat at 55°, Woven Roving at −55°, Mat at 55°, Mat at −55°, Woven Roving at 55°, and finally, Mat at −55°. The thickness considered for the Woven Roving fiber is 1 mm and for the Mat fiber is 0.8 mm.

**Figure 5:**
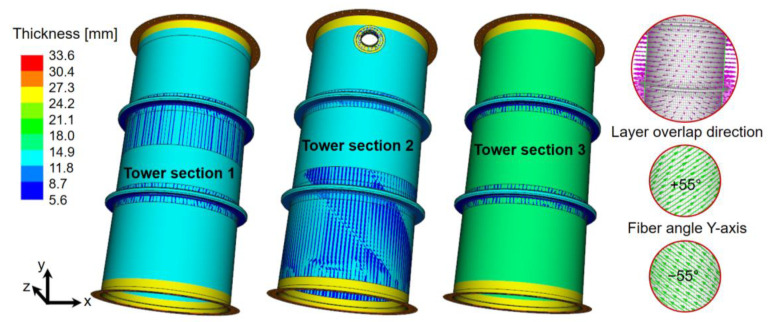
Thickness and lamination of gas absorption tower sections.

The thicknesses specified in Table 1 were applied to the digital models of the absorption tower sections in the ACP Pre module of ANSYS. Figure 5 shows the three sections of the gas absorption tower, along with their respective laminate thicknesses. The areas where blue lines are observed in the shell correspond to the structural reinforcement bands.

Based on the lamination of the absorber tower sections, the masses of each of the three tower sections were estimated as 552, 547, and 563 kg, respectively. For simplicity and for a more conservative assumption, the deadweight values applied in the modeling of each section are set as 600 kg.

### 2.5. Mesh Sensitivity Analysis of Tower Sections and Components in Structural Analysis

A mesh convergence study was conducted to ensure accurate results in areas with high stress and strain gradients. The study involved comparing geometries in four different zones (the upper flange, the flange edge notch, the tower section shell, and the ceramic packing support ring) and using both plate and solid elements. The goal was to achieve convergence by reducing the number of mesh elements while still obtaining reliable results. By optimizing the reduction of elements, the study aimed to find the minimum number of elements necessary for accurate analysis.

The meshing study considered the bores as fixed and the load of the ceramic packing of 50 kN was applied in the support ring. The structural analysis for the lifting operation was performed separately for each section, applying the corresponding loads and constraints. The sizing tool was applied to control the mesh dimensions in these zones. The study compared plate and solid elements, in order to reduce the number of mesh elements and to be able to use the Ansys software with a student version.

### 2.6. Modeling Loading Conditions of the Lifting Operation

The gas absorption tower sections must be capable of being transported from one location to another by vertical or horizontal hoisting. Therefore, both cases must be studied for the three existing sections. Given the conditions of a hoisting, which require a gradual movement at a very slow speed, a static-structural study will be considered. The loading conditions for the two lifting options are described below.

**Figure 6:**
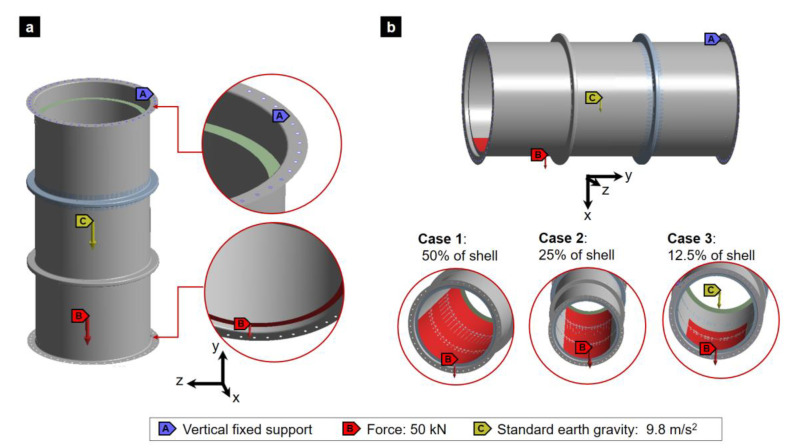
Loading conditions: (**a**) vertical lifting and (**b**) horizontal lifting with ceramic packing load on different percentages of the inner surface.

- Vertical lifting: For the case of vertical lifting, the first condition considered was the fastening of the tower by bolts, which will go in their respective bolt circle of the upper flange. Therefore, the ANSYS Fixed Support function was used, which fixes these holes. Additionally, a force corresponding to 5000 kg of packing ceramics, equivalent to 50 kN, was applied on the ceramic packing support ring with a direction on the *Y*-axis, coinciding with the direction of gravity acceleration. In Figure 6a, the loading conditions for the study of vertical lifting are shown.
- Horizontal lifting: For the case of horizontal lifting, the absorption tower sections were considered to be bolted at their two flanges. Vertical fixed support was used in the holes of both flanges. The gravity force was applied in the X direction. Finally, different ceramic packing was modeled as a distributed load of 50 kN for three different cases: case 1 loaded in 50% of the shell, case 2 in 25%, and case 3 in 12.5% of the shell inner surface.

## 3. Analysis of Results and Discussion

### 3.1. Sensitivity Analysis of Mesh: Effect of Mesh Size on Tower Stress Field Convergence

**Figure 7:**
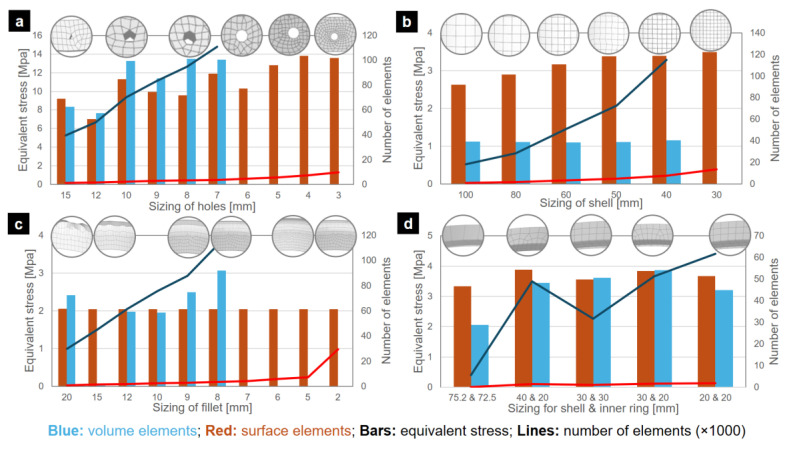
Mesh convergence study results: (**a**) flange with holes, (**b**) shell (mantle), (**c**) fillet, and (**d**) inner ring and shell.

The mesh convergence study considering plate and solid elements was performed with specific mesh sizes in different sections to optimize the achieved reliable results in critical areas with high stress and strain gradients with the lowest number of elements. The mesh convergence analysis yielded the following results (Figure 7):

- For flange edges with holes, a sizing was assigned to the holes. The volumetric geometry tends to reach an equivalent stress of 13.37 MPa with a quantity of 94 thousand elements or higher, using a sizing of 8 mm. In surface geometry, it is possible to reach a similar value of 12.79 MPa with only 5675 elements, equivalent to a sizing of 5 mm.
- For tower shells, a general sizing is applied to this zone. For the volumetric geometry, although a smaller sizing will be assigned, its stress value did not vary significantly, remaining around 1.1 MPa, even considering that the number of elements was increased to 115 thousand elements. In the case of surface geometry, the equivalent stress varied from 2.63 MPa to 3.49 MPa. The case with the largest number of elements had only 13,536 elements for a 30 mm sizing, considering that its predecessor, the 40 mm sizing, has only 7668 elements.
- For notches, a sizing was assigned specifically to the notch curvature. In the volumetric geometry, over 87 thousand elements in the case of 9 mm sizing, an increase in the equivalent stress was observed, going from 1.95 MPa with 10 mm sizing to 3.06 MPa with 8 mm sizing, reaching a quantity of 112 thousand elements. On the other hand, the surface geometry was maintained with an average of 2 MPa and the number of elements remained under 8 thousand until the sizing of 5 mm.
- For the joint between the support ring of ceramic packing and the shell, a sizing was applied to the ring and to the corresponding shell zone, evaluating several combinations. For the volumetric case, the values tend to be around 3.6 MPa, and the number of elements varies depending on the combination of sizing in the ring and the shell, always exceeding 31 thousand elements for the cases with results similar to 3.6 MPa. For the surface geometry case, it is possible to obtain results similar to those of the volume geometry with the default ANSYS sizing configuration, which is 75.2 mm, achieving 3.34 MPa with only 176 elements.

Based on the mesh convergence study and to reduce the number of elements, the lifting study in the gas absorption tower sections was performed with surface geometries. In addition, considering the junction of the different zones and the need to avoid abrupt changes between the different meshing areas, the sizing distribution was decided according to Table 3.

**Figure 8:**
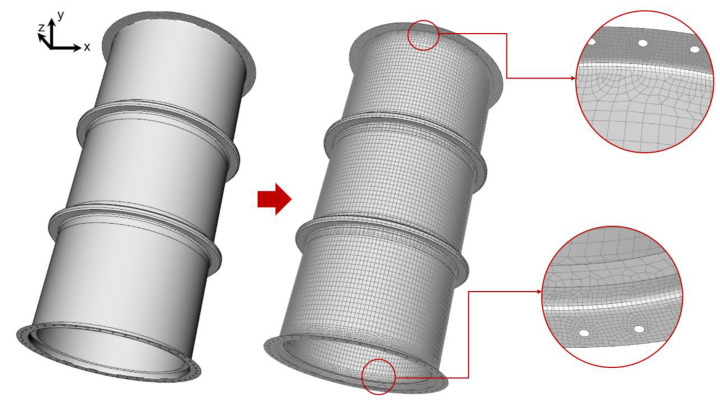
Meshing representation in section (3) of the gas absorption tower.

To accommodate varying thicknesses within the absorption tower sections, face meshing was employed on both the shell and fillets. This technique effectively defines the zones of interest. Figure 8 illustrates the meshing applied to tower section (3).

### 3.2. Stress Resistance and Damage of Tower Sections during Vertical Hoisting

In the case of vertical lifting, the unit deformations are low, in the order of 0.0006, so this study will focus on the equivalent stresses of the absorption tower sections and the Hashin failure criterion.

**Figure 9:**
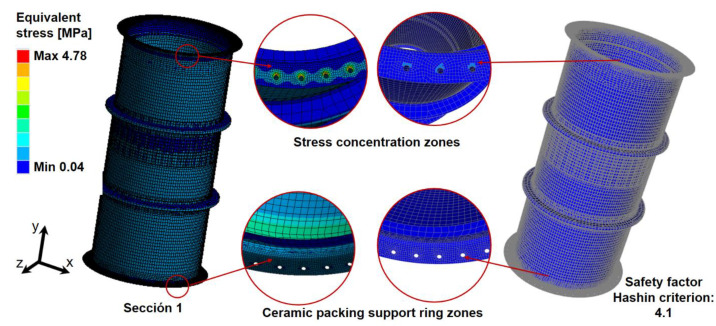
Structural analysis of GFRP tower section 1 during vertical lifting: (stress distribution and the factor of safety.

For section (1), the maximum equivalent stress occurs in the bolt zone, with a value of 4.77 MPa. Subsequently, in the ACP post analysis, the failure criterion for that zone indicates a factor of safety of 4.1 is reached. The distribution of equivalent stress and factor of safety according to Hashin’s criterion from the ACP Post module is presented in Figure 9.

**Figure 10:**
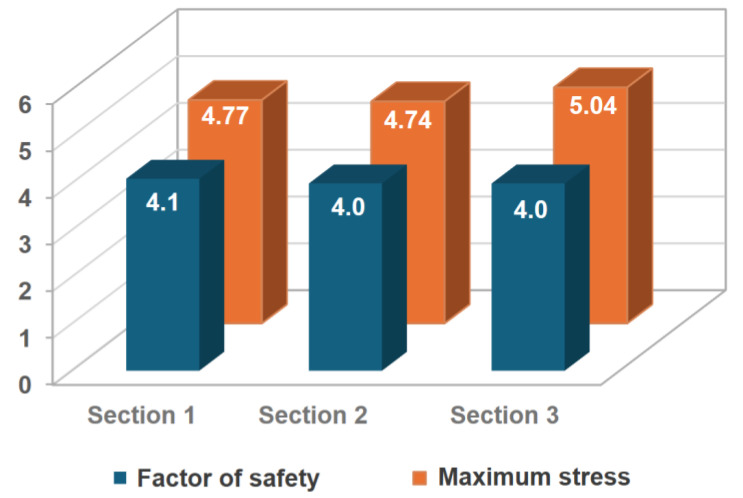
Comparative results of the vertical lifting structural study for the three sections.

Sections (2) and (3) show similar trends of stress distributions, with maximum equivalent stresses of 4.74 MPa and 5.04 MPa, respectively. The factors of safety remained at 4.0for both sections. Figure 10 shows the summary of results for the structural analysis in vertical lifting for the three sections of the gas absorption tower.

### 3.3. GFRP Tower Response to Horizontal Hoisting: Influence of Ceramic Packing Weight

**Figure 11:**
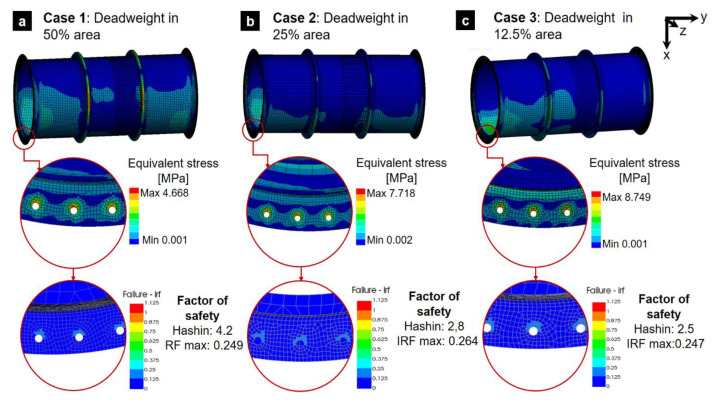
Stresses and the factor of safety using Hashin’s failure criterion for the horizontal hoist of section (1).

This analysis investigates the structural performance of a GFRP tower during horizontal hoisting, with a focus on how the distribution of ceramic packing weight affects stress levels and safety factors. Due to minimal unit deformations (maximum 0.00013), the study prioritized analyzing equivalent stresses using Hashin’s failure criterion, which is well-suited for GFRP composite materials. Figure 11 depicts the stress distribution for each load case in section (1). The three different simulated ceramic packing distributions showed that the maximum equivalent stress consistently occurred at the bolt holes (stress concentration zone). The results are summarized below:

- Case 1: An evenly distributed load resulted in a maximum stress of 4.66 MPa and a factor of safety of 4.2.
- Case 2: A more concentrated load distribution led to a higher maximum stress of 7.71 MPa and a reduced factor of safety of 2.8.
- Case 3: The most unfavorable scenario, with packing concentrated over 12.5% of the shell, yielded the highest stress of 8.74 MPa and a factor of safety of 2.5.

**Figure 12:**
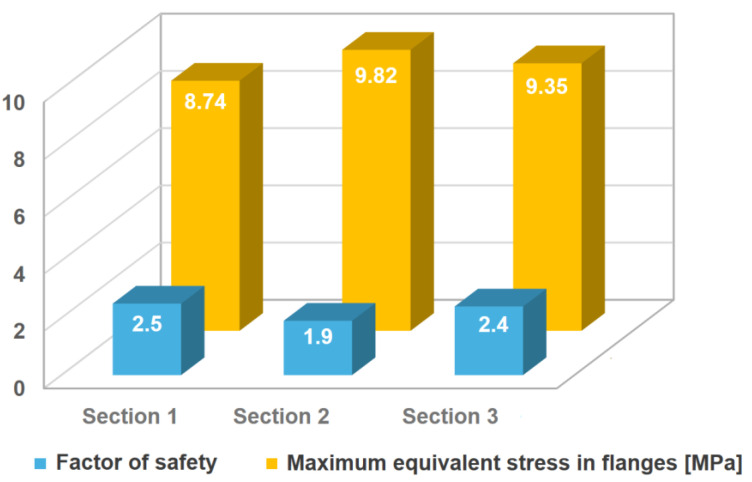
Comparative results of horizontal lifting simulation for the three tower sections.

Given the high stress observed in Case 3 of section (1), the analysis was extended to sections (2) and (3) using the same unfavorable load distribution. This revealed maximum stresses of 9.82 MPa and 9.35 MPa for sections (2) and (3), respectively. The corresponding factors of safety were 1.9 and 2.4. Figure 12 provides a comparative visualization of these results. Note that even if several adequate assumptions to model the real case were made and reliable numerical models were developed based on mesh convergence criteria, materials properties were considered as informed by industry standards. The experimental validation of hoisting operations is not common prior to areal case application. The required hoisting process and design can be verified using adapted strain gage devices located in critical positions of the section tower during the hoisting operation. By using adequate models, damage can be verified. While the analysis indicates the structural feasibility of both vertical and horizontal lifting for the tower sections, the low safety factors observed in certain scenarios, particularly horizontal lifting with concentrated packing weight, necessitate a more detailed investigation. A comprehensive assessment, encompassing industry standards, dynamic loading effects, and potential mitigation strategies, is crucial to ensure long-term structural integrity. Furthermore, recognizing the potential degradation of material properties due to environmental factors such as temperature cycles and exposure to water or other media [12], future research should account for these variables.

To ensure the accuracy and reliability of numerical simulations for the GFRP section tower in lifting operations, it is important to conduct comprehensive experimental validation. This can be done by using strain gage devices placed at critical positions of the tower sections during the actual lifting process. By comparing the measured strain and stress distributions with the values predicted by the numerical models, any discrepancies can be identified and the models can be refined accordingly. The numerical models used in the study were developed based on appropriate assumptions and material properties from industry standards, but experimental validation is necessary to verify their accuracy. Moreover, experimental validation can evaluate the tower’s behavior under dynamic loading conditions, which were not fully accounted for in the study. By monitoring the tower’s response during the lifting process, unexpected stress concentrations or deformations can be detected and included in the analysis and final design of hoisting elements.

## 4. Conclusions

The key conclusions from this structural integrity analysis of the GFRP absorption tower sections for horizontal and vertical lifting operations are as follows:

♦ Surface geometry models can achieve comparable stress results to more computationally intensive volumetric models, but with significantly fewer elements, reducing computational resources required.
♦ The analysis of the lifting operations for the FRP tanks demonstrated factors of safety ranging from 1.9 to 2.5 for horizontal lifting and a factor of safety of 4.0 for vertical lifting, providing a sufficient margin for safe execution.
♦ However, low safety factors were observed in certain scenarios, especially for horizontal lifting with concentrated packing weight, necessitating a comprehensive assessment considering industry standards, dynamic loads, potential material degradation, and mitigation strategies to ensure long-term structural integrity.

Future work should involve experimentally validating the numerical simulations of GRP tower sections during lifting operations and carefully designing the hoisting elements to ensure overall safety and reliability.

## Figures and Tables

**Table 1:** Lamination by zone of the gas absorption tower sections.

Section Area	Section (1)	Section (2)	Section (3)	Thickness (mm)
Upper and lower flange	10 MWM	10 MWM	10 MWM	28
Top and bottom trim	9 MWM	9 MWM	9 MWM	25.2
Tower shell	5 MWM	5 MWM	5 MWM + 2 M	14 (sections (1) and (2)) 16 (section (3))
Additional layers to the shell		1 M (from bottom edge to 1258 mm to top edge)	2 M	1
Structural reinforcements: inner radius and joint to 788 mm radius	2 MWM	2 MWM	2 MWM	5.6
Structural reinforcements: 788 mm radius only	4 MWM + M	4 MWM + M	4 MWM + M	12.2
Structural band	MWM	-	-	2.8
Shell over-thickness	-	1 M	2 M	1 M
Pass-through nozzle	-	7 MWM	-	19.6
Inner nozzle	-	3 M	-	8.4
External splice	-	9 MWM	-	25.2
Internal splices	-	4 M	-	11.2

**Table 2:** Mechanical properties of Woven Roving and Mat glass-fiber reinforced polymer composites.

			GFRP Fiber Type	
Property	Direction	Units	Woven Roving	Mat
Density		[kg/m^3^]	1850	1508.6
Tensile strength	x	[MPa]	487.1	106.03
	y		487.1	106.03
	z		7.7	31
Compressive strength	x		−292.3	−141.3
	y		−292.3	−141.3
	z		−41.2	−100
Shear strength	xy		49.6	71.3
	yz		35	35
	xz		35	35
Young’s modulus	x		24,355	7069
	y		24,355	7069
	z		5154.6	5268
Shear modulus	xy		1895	2663
	yz		1532	2152
	xz		1532	2152
Poisson’s ratio	xy	Unitless	0.3011	0.3269
	yz		0.28	0.28
	xz		0.28	0.28

**Table 3:** Sizing of the mesh refinement zones of the studied tower sections.

Refinement Zone	Sizing (mm)
Hole circle	5
Fillet	9
Ring and shell joints	30 & 30
Shell	40

## Data Availability

The raw data supporting the conclusions of this article will be made available by the authors upon request.
